# Central venous pressure measurement is associated with improved outcomes in septic patients: an analysis of the MIMIC-III database

**DOI:** 10.1186/s13054-020-03109-9

**Published:** 2020-07-14

**Authors:** Hui Chen, Zhu Zhu, Chenyan Zhao, Yanxia Guo, Dongyu Chen, Yao Wei, Jun Jin

**Affiliations:** 1 Department of Critical Care Medicine, The First Affiliated Hospital of Soochow University, Suzhou, 215000 Jiangsu China; 2 Department of General Surgery, The Affiliated Suzhou Science & Technology Town Hospital of Nanjing Medical University, Suzhou, 215000 Jiangsu China; 3 Department of Intensive Care Medicine, Yancheng City No.1 People’s Hospital, Yancheng, 224000 Jiangsu China; 4 Department of Intensive Care Medicine, The First Affiliated Hospital of Soochow University, No. 899 Pinghai Road, Suzhou, 215000 Jiangsu China

**Keywords:** Central venous pressure, Sepsis, Lactate, 28-day mortality

## Abstract

**Purpose:**

Measurement of central venous pressure (CVP) can be a useful clinical tool. However, the formal utility of CVP measurement in preventing mortality in septic patients has never been proven.

**Methods:**

The Medical Information Mart for Intensive Care III (MIMIC-III) database was searched to identify septic patients with and without CVP measurements. The primary outcome was 28-day mortality. Multivariate regression was used to elucidate the relationship between CVP measurement and 28-day mortality, and propensity score matching (PSM) and an inverse probability of treatment weighing (IPTW) were employed to validate our findings.

**Results:**

A total of 10,275 patients were included in our study, of which 4516 patients (44%) underwent CVP measurement within 24 h of intensive care unit (ICU) admission. The risk of 28-day mortality was reduced in the CVP group (OR 0.60 (95% CI 0.51–0.70; *p* < 0.001)). Patients in the CVP group received more fluid on day 1 and had a shorter duration of mechanical ventilation and vasopressor use, and the reduction in serum lactate was greater than that in the no CVP group. The mediating effect of serum lactate reduction was significant for the whole cohort (*p* = 0.04 for the average causal mediation effect (ACME)) and patients in the CVP group with an initial CVP level below 8 mmHg (*p* = 0.04 for the ACME).

**Conclusion:**

CVP measurement was associated with decreased risk-adjusted 28-day mortality among patients with sepsis and was proportionally mediated through serum lactate reduction.

## Introduction

Sepsis is a major challenge in intensive care unit (ICU) settings and accounts for approximately 30 to 50% of short-term mortality [1, 2]. Haemodynamic monitoring plays a critical role in the management of sepsis. As a component of early goal-directed therapies (EGDTs), central venous pressure (CVP) has been utilized to predict status or fluid responsiveness [3]; however, the most recent guideline no longer recommended CVP to guide fluid administration in septic patients [4]. This statement discounts the physiological value of CVP in clinical. With proper insights, the measurement of CVP can be a useful clinical tool.

Fluid administration is a double-edged sword; the benefits of fluid administration include an increase in cardiac output, but the risks include an increase in hydrostatic pressure, increasing oedema formation. It is more beneficial and less risky to administer fluids in patients with a lower CVP than in those with a high CVP. In a study that included 556 ventilated ICU patients, a positive response to fluid was observed when CVP values were less than 6 mmHg but not when values were greater than 15 mmHg [5]. A retrospective study conducted by Legrand declared that a high CVP value within the first 24 h of admission was associated with the risk of developing new or persistent acute kidney injury (AKI) [6], and limiting CVP in liver surgery is associated with a decreased risk of bleeding and improved perioperative outcomes [7]. Furthermore, the CVP waveform provides information about inspiratory effort, chest wall and right ventricular compliance and the likelihood of cardiac tamponade [8]. There are limited studies focused on CVP measurement and its effect on outcomes. In a prospective randomized controlled trial [9], CVP measurement and oesophageal Doppler ultrasonography shortened the time to being declared medically fit for discharge in patients undergoing proximal femoral fracture repair under general anaesthesia. Another study demonstrated that a goal-directed protocol using CVP, mean arterial pressure (MAP) and urine output values as therapeutic goals improved survival and clinical outcomes in patients with septic shock [10]. However, the formal utility of CVP measurement in predicting mortality in septic patients has never been proven.

The mediating effects of CVP measurement on mortality are equally important. Therapeutic interventions guided by CVP measurement are aimed at providing adequate oxygen availability and revising tissue hypoperfusion [11]. An elevated lactate level in septic patients is a predictor of poor clinical outcome and a biomarker of tissue hypoperfusion, and a reduction in lactate levels seems to be associated with reduced mortality in critically ill patients [12]. Causal mediation analysis (CMA) [13] was used to investigate the mediating effects of lactate reduction on CVP measurement in terms of mortality.

In the present study, we aimed to elucidate the effect of CVP measurement on 28-day mortality in septic patients. We hypothesized that CVP measurement was associated with lower 28-day mortality and proportionally mediated through a reduction in lactate.

## Methods

### Study design

We conducted a retrospective cohort study based on a large US-based database called the Medical Information Mart for Intensive Care III (MIMIC-III) [14]. The MIMIC-III (v1.4) database contains comprehensive and high-quality data of well-defined and characterized ICU patients admitted to ICUs at the Beth Israel Deaconess Medical Center between 2001 and 2012. One author (HC) obtained access to the database and was responsible for data extraction (certification number 27252652). Our study complied with the Reporting of Studies Conducted using Observational Routinely Collected Health Data (RECORD) statement [15].

### Selection of participants

Patients in the MIMIC-III who fulfilled the definition of sepsis were eligible for inclusion. Sepsis was diagnosed according to the sepsis-3 criteria [16]; in brief, patients with documented or suspected infection and an acute change in total Sequential Organ Failure Assessment (SOFA) score of ≥ 2 points were considered to have sepsis. Infection was identified from the International Classification of Diseases 9th Edition (ICD-9) code in the MIMIC-III. We excluded patients who were younger than 18 years or who spent less than 24 h in the ICU. Additionally, we analyzed only the first ICU stay for patients who were admitted to the ICU more than once. Included patients for whom initial CVP measurements were completed within 24 h after ICU admission were classified as the CVP group, and the rest of the patients comprised the no CVP group.

### Variable extraction

The primary exposure was whether the patients underwent CVP measurements. The time to initial CVP measurement, the initial level of CVP and the duration of use of CVP were also collected. Baseline characteristics within the first 24 h after ICU admission were collected using structured query language (SQL), including age, sex, weight, ICU type, severity at admission as measured by SOFA score, the Simplified Acute Physiology Score II (SAPS II) and the Elixhauser comorbidity score. The use of mechanical ventilation, application of renal replacement therapy (RRT), and administration of vasopressors were also recorded. Vital signs included the MAP, heart rate, temperature (°C) and respiratory rate. Laboratory variables including white blood cell (WBC) count, haemoglobin, platelet counts, lactate, pH, partial pressure of oxygen (PO_2_) and partial pressure of carbon dioxide (PCO_2_) were measured during the first 24 h in the ICU. If a variable was recorded more than once in the first 24 h, we used the value related to the greatest severity of illness. The incidence of AKI was also extracted, and AKI was defined according to the Kidney Disease Improving Global Outcomes (KDGIO) criteria.

Comorbidities including congestive heart failure (CHF), atrial fibrillation (AFIB), chronic renal disease, liver disease, chronic obstructive pulmonary disease (COPD), stroke and malignant tumour were also collected for analysis based on the recorded ICD-9 codes in the MIMIC-III database.

### Outcomes

The primary outcome in the present study was 28-day mortality. Secondary outcomes included in-hospital and 1-year morality; the incidence of AKI within 7 days after ICU admission; the volumes (L) of intravenous fluid (IVF) in the first, second and third days in the ICU; the number of ventilator-free and vasopressor-free days within 28 days after ICU admission; and reduction in serum lactate (calculated as the difference between the maximum lactate level on day 1 and day 3).

### Statistical analysis

Values are presented as the means (standard deviations) or medians [interquartile ranges (IQRs)] for continuous variables, and categorical variables are presented as total numbers and percentages. Comparisons between groups were made using the *X*^2^ test or Fisher’s exact test for categorical variables and Student’s *t* test, or the Mann-Whitney *U* test for continuous variables, as appropriate.

Multivariate regression was selected to characterize the relationship between CVP measurement and the primary outcome. Baseline variables that were considered clinically relevant or that showed a univariate relationship with the outcome (*p* < 0.10), including age, sex, weight admission period, severity score, use of mechanical ventilation, use of RRT, use of vasopressors, comorbidities, AKI, vital signs (MAP, heart rate, temperature and respiratory rate) and initial lactate level, were entered into a multivariate logistic regression model as covariates. To avoid bias induced by missing data, the analysis of the primary outcome was duplicated after multiple imputations.

Propensity score matching (PSM) and propensity score-based inverse probability of treatment weighing (IPTW) were also used to adjust the covariates to ensure the robustness of our findings [17, 18]. A multivariate logistic regression model was used to estimate the patient’s propensity scores for CVP measurement. One-to-one nearest neighbour matching with a calliper width of 0.05 was applied in the present study. An IPTW model was created using the estimated propensity scores as weights. The standardized mean differences (SMDs) were calculated to evaluate the effectiveness of the PSM and IPTW. Logistic regression was then performed on the matched cohort and weighted cohort, separately. Outcomes and therapeutic interventions were generated from the matched cohort.

CMA is a method to differentiate the total effect of a treatment into direct and indirect effects. The indirect effect on the outcome is mediated via a mediator. The analysis produces an average causal mediation effect (ACME), average direct effect (ADE) and total effect. To explore whether the effect of CVP measurement on the primary outcome is proportionally mediated by the reduction in serum lactate, we used CMA to characterize the causality relationship in our retrospective study.

Subgroups analyses of patients with positive blood cultures and septic shock were performed. Given that the effect of CVP measurement may vary according to the duration of CVP measurement and the initial CVP level, we first performed a series of multivariate logistic regressions based on the different duration of CVP measurement (1 day, 2 days, 3 days, 4 days, 5 days, 6 days, 7 days and > 7 days), and then conducted sensitivity analyses comparing patients whose initial CVP level was below 8 mmHg or above 15 mmHg with patients in the no CVP group to evaluate the robustness of our findings.

All statistical analyses were performed using RStudio (version 1.2.5019), and *p* < 0.05 was considered statistically significant.

## Results

### Baseline characteristics

**Fig. 1:**
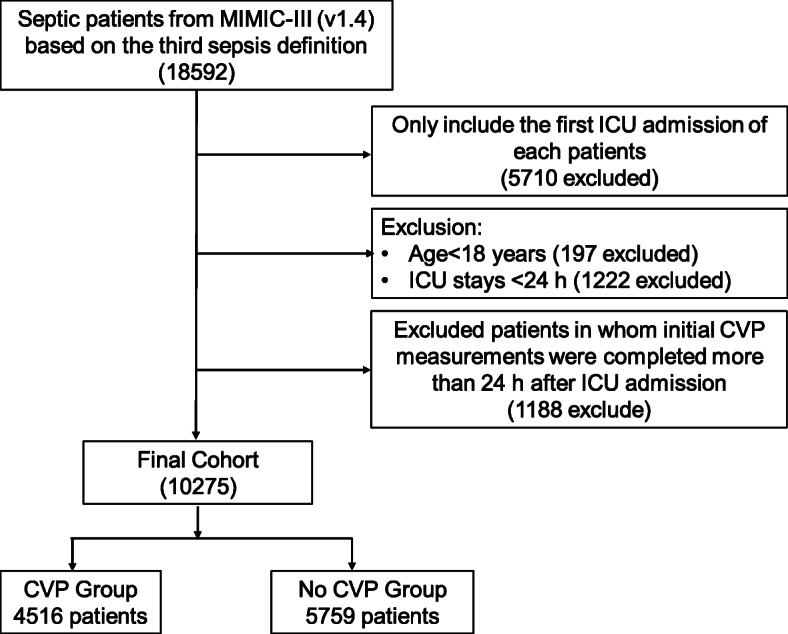
Study flow diagram in the present study

After reviewing the data of 18,592 septic patients, a total of 10,275 patients were included in our study. The flow diagram of patient selections is presented in Fig. 1. Among the study cohort, 4516 patients (44%) had their CVP measured within 24 h after ICU admission, with a median value of 11 mmHg (IQR, 8–15 mmHg); the duration of use of CVP was 2.9 days (IQR, 1.3–7.4 days); and the time to initial CVP measurement was 3 h (IQR, 1.6–7.8 h) (Additional file 1: Fig. S1). The baseline characteristics of the CVP and no CVP groups are summarized in Table 1. Patients in the CVP group had significantly higher SOFA scores (6 (4–9) vs. 4 (3–6)) and lactate levels (1.9 mmol/L (IQR, 1.3–3.1 mmol/L) vs. 1.5 mmol/L (IQR, 1.1–2.1 mmol/L)) on admission than those in the no CVP group. Within the first 24 h after ICU admission, the CVP group was more likely to receive mechanical ventilation (74.5 vs. 34.2%) and vasopressors (31.1 vs. 4.3%) than the no CVP group.

**Table 1:** Comparisons of baseline characteristics between the original cohort and matched cohort

Covariates	Original cohort			Matched cohort		
	CVP	No CVP	SMD	CVP	No CVP	SMD
*N*	4516	5759		2174	2174	
Age	69 (56–79)	71 (56–82)	0.079	69 (56–80)	69 (55–81)	0.003
Male (%)	2473/4516 (54.8)	2931/5759 (50.9)	0.078	1131/2174 (52)	1166/2174 (53.6)	0.032
Weight (kg)	79.5 (66.8–95)	75 (62.6–90.2)	0.168	78 (65–94)	77 (64.5–92.5)	0.020
Service unit, *n* (%)			0.675			0.036
MICU	1811/4516 (40.1)	3251/5759 (56.5)		1113/2174 (51.2)	1095/2174 (50.4)	
SICU/TSICU	1213/4516 (26.9)	1451/5759 (25.2)		637/2174 (29.3)	646/2174 (29.7)	
CCU/CSRU	1492/4516 (33.0)	1057/5759 (18.3)		424/2174 (19.5)	433/2174 (19.9)	
Admission period, *n* (%)			0.273			0.004
Before 2008	2854/4516 (63.2)	2868/5759 (49.8)		1307/2174 (60.1)	1311/2174 (60.3)	
2008–2012	1662/4516 (36.8)	2891/5759 (50.2)		867/2174 (39.9)	863/2174 (39.7)	
Severity of illness						
SOFA score	6 (4–9)	4 (3–6)	0.795	5 (4–7)	5 (3–7)	0.018
SAPS II score	43 (34–54)	37 (30–46)	0.465	41 (32–49)	40 (32–50)	0.008
Elixhauser comorbidity score	7 (3–12)	7 (2–12)	0.025	7 (3–13)	7 (3–13)	0.013
Interventions, *n* (%)						
MV use (1st 24 h)	3366/4516 (74.5)	1971/5759 (34.2)	0.885	1189/2174 (54.7)	1203/2174 (55.3)	0.013
Vasopressor use (1st 24 h)	1406/4516 (31.1)	246/5759 (4.3)	0.752	259/2174 (11.9)	236/2174 (10.9)	0.033
RRT use (1st 24 h)	238/4516 (5.3)	280/5759 (4.9)	0.019	116/2174 (5.3)	104/2174 (4.8)	0.025
Comorbidities, *n* (%)						
CHF	996/4516 (22.1)	1296/5759 (22.5)	0.011	535/2174 (24.6)	551/2174 (25.3)	0.002
AFIB	990/4516 (21.9)	1428/5759 (24.8)	0.068	535/2174 (24.6)	527/2174 (24.2)	0.009
Chronic renal disease	608/4526 (13.5)	1129/5759 (19.6)	0.166	334/2174 (15.4)	329/2174 (15.1)	0.006
Liver disease	405/4526 (9.0)	440/5759 (7.6)	0.048	183/2174 (8.4)	183/2174 (8.4)	< 0.001
COPD	908/4526 (20.1)	1272/5759 (22.1)	0.049	477/2174 (21.9)	458/2174 (21.1)	0.021
Stroke	151/4526 (3.3)	231/5759 (4.0)	0.035	90/2174 (4.1)	83/2174 (3.8)	0.016
Malignancy	152/4526 (3.4)	201/5759 (3.5)	0.007	73/2174 (7.4)	76/2174 (3.5)	0.008
Vital signs						
MAP (mmHg)	54 (48–60)	58 (51–65)	0.358	55 (49–62)	56 (49–63)	0.013
Heart rate (bpm)	108 (94–124)	104 (90–119)	0.229	107 (92–122)	106 (93–121)	0.001
Temperature (°C)	37.7 (37.2–38.3)	37.4 (37–38)	0.260	37.6 (37–38.2)	37.6 (37–38.2)	< 0.001
Respiratory rate (bpm)	28 (24–33)	27 (24–32)	0.102	28 (24–33)	28 (24–32)	0.004
Laboratory tests						
WBC (× 10^9^/L)	15.2 (10.8–20.8)	12.2 (8.7–16.9)	0.253	14.5 (10.2–19.7)	13.1 (9.3–18.2)	0.046
Hemoglobin (× 10^12^/L)	9.2 (8–10.5)	10.2 (8.9–11.6)	0.486	9.7 (8.5–11)	9.6 (8.4–11)	0.030
Platelet (× 10^9^/L)	158 (105–231)	197 (133–270)	0.277	186 (126–257)	187 (118–261)	0.028
Bicarbonate (mmol/L)	21 (18–23)	23 (19–26)	0.435	21 (18–24)	21 (18–24.8)	0.002
Bun (mg/dL)	26 (17–43)	26 (17–42)	0.023	27 (17–44)	26 (17–44)	0.004
Creatinine (mg/dL)	1.3 (0.9–2.1)	1.2 (0.8–1.9)	0.009	1.2 (0.9–2.0)	1.2 (0.8–2.0)	0.010
Lactate level (mmol/L)	1.9 (1.3–3.1)	1.5 (1.1–2.1)	0.419	1.6 (1.2–2.4)	1.6 (1.1–2.4)	0.024
pH	7.36 (7.32–7.40)	7.40 (7.36–7.44)	0.535	7.38 (7.33–7.42)	7.38 (7.34–7.43)	0.010
pO_2_ (mmHg)	131 (98–178)	109 (84–146)	0.345	117 (90–156)	117 (90–157)	0.040
pCO_2_ (mmHg)	39 (35–43)	39 (35–44)	0.132	39 (35–44)	39 (34–44)	0.004
AKI, *n* (%)	3488/4526 (77.2)	3502/5759 (60.8)	0.361	1507/2174 (69.3)	1489/2174 (68.5)	0.018

*MICU* medical intensive care, *SICU* surgical intensive care unit, *TSICU* trauma surgical intensive care unit, *CCU* coronary care unit, *CSRU* cardiac surgery unit, *SOFA* Sequential Organ Failure Assessment, SAPS II Simplified Acute Physiology Score II, MV mechanical ventilation, *RRT* renal replacement therapy, *CHF* congestive heart failure, *AFIB* atrial fibrillation, *COPD* chronic obstructive pulmonary disease, *MAP* mean arterial pressure, *WBC* white blood cell, *PO*_*2*_ partial pressure of oxygen, *PCO*_*2*_ partial pressure of carbon dioxide, *AKI* acute kidney injury

### Primary outcome

**Fig. 2:**
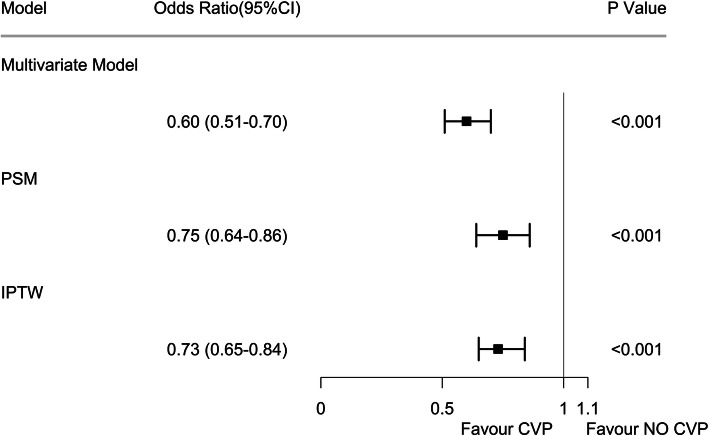
Association between CVP measurement and 28-day mortality. The odds ratios and 95% confidence intervals (error bars) in both cohorts were calculated dependent on the method of covariate adjustment. PSM, propensity score matching; IPTW, inverse probability of treatment weight

The multivariate logistic regression analyses showed a significant beneficial effect of CVP measurement in terms of 28-day mortality (Fig. 2), with an adjusted odds ratio (OR) of 0.60 (95% CI 0.51–0.70). The results were maintained after multiple imputations for missing values (Additional file 1: Table S1 and S2). After PSM and IPTW, the imbalance in the covariates between the CVP and no CVP groups was significantly minimized (Additional file 1: Fig. S2), and the association remained robust (Fig. 2). We also performed subgroup analyses to investigate patients with positive blood cultures and septic shock, and both subgroup analyses produced the same result (Additional file 1: Table S3 and S4).

### Secondary outcomes with propensity score-matched cohorts

**Fig. 3:**
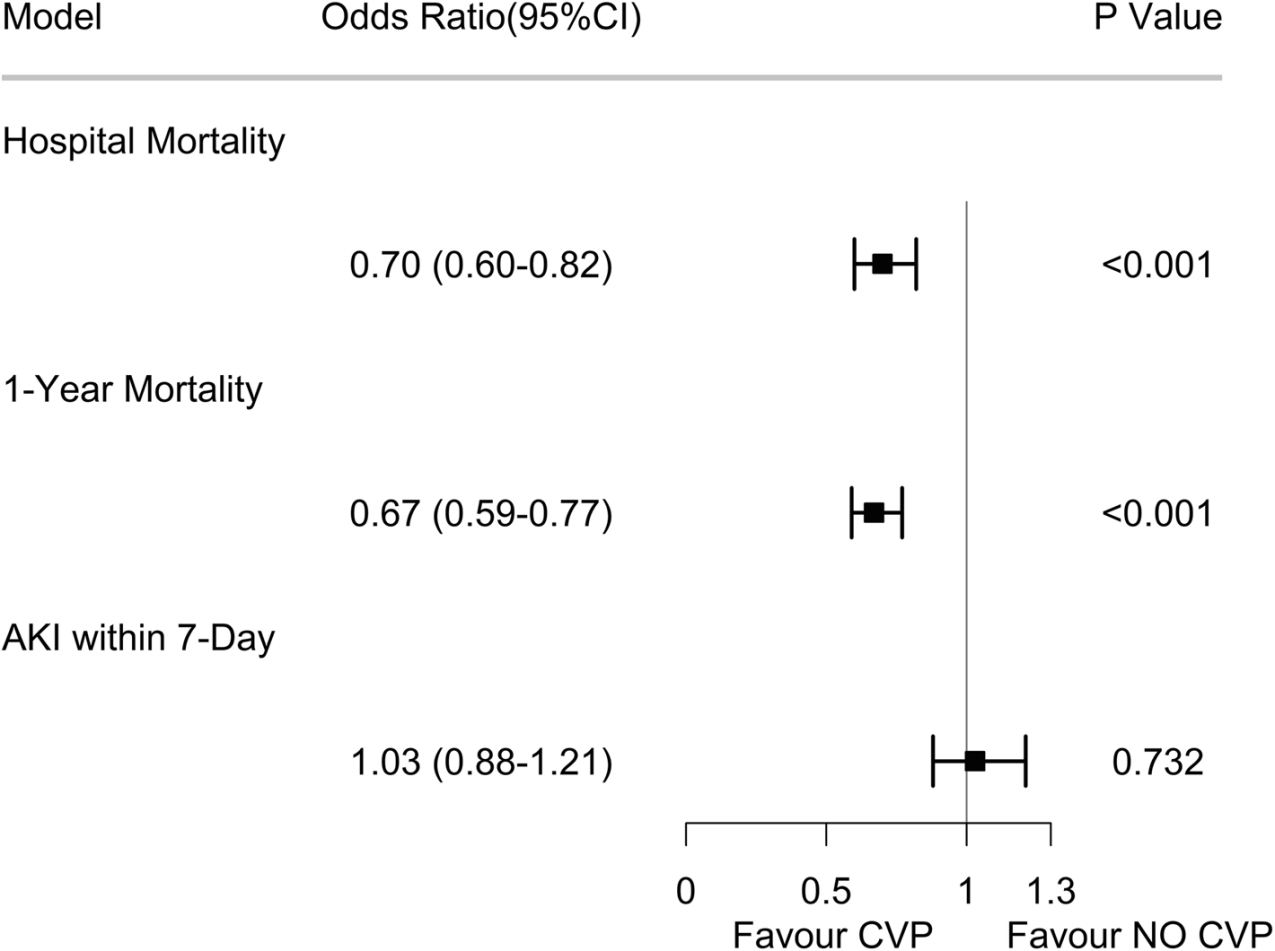
Association between CVP measurement and secondary outcomes. The odds ratios and 95% confidence intervals (error bars) in both cohorts were calculated from the multivariable logistic regression. AKI, acute kidney injury

CVP measurement was also associated with lower risk-adjusted in-hospital mortality and 1-year mortality, but not with AKI within 7 days after ICU admission (Fig. 3). Numerous therapeutic interventions that might account for the beneficial effects of CVP measurement were also investigated. Compared with the no CVP group, the volume of IVF in the CVP group was significantly higher on day 1 (2.4 vs. 1.9 L; *p* < 0.001), but there were no differences on day 2 and day 3. Patients in the CVP group had shorter durations of mechanical ventilation and vasopressor use than patients in the no CVP group. With respect to lactate, we observed that the reduction in serum lactate between day 1 and day 3 was higher in the CVP group than in the no CVP group (1.48 vs. 1.13 mmol/L; *p* = 0.029). Table 2 shows the detailed results.

**Table 2:** Clinical outcomes analysis with propensity score matched cohorts

Outcomes	CVP	NO CVP	Effect size	*p* value
**Primary outcome**				
28-day mortality	396/2174 (18.2)	500/2174 (23)	0.118	< 0.001
**Secondary outcomes**				
In-hospital mortality	366/2174 (16.8)	415/2174 (19.1)	0.059	0.058
1-year mortality	789/2174 (36.3)	920/2174 (42.3)	0.124	< 0.001
AKI within 7 days, *n* (%)	1722/2174 (79.2)	1692/2174 (77.8)	0.029	0.355
Volume of IVF on day 1(mL)	2380 (1037–4245)	1897.5 (890–3070)	0.327	< 0.001
Volume of IVF on day 2 (mL)	997 (289.2–2150)	1000 (268.25–1953.6)	0.142	0.054
Volume of IVF on day 3 (mL)	605 (240–1500)	625 (230–1580.5)	0.051	0.942
Vasopressor-free day in 28 days	26.6 (25–27.4)	26.2 (22.9–27.2)	0.472	< 0.001
Ventilation-free day in 28 days	25.8 (22.3–27.1)	23.3 (17.3–26.2)	0.291	< 0.001
Delta-lactate	1.48 (2.35)	1.13 (2.32)	0.148	0.029

*IVF* intravenous fluid, *AKI* acute kidney injury

**Fig. 4:**
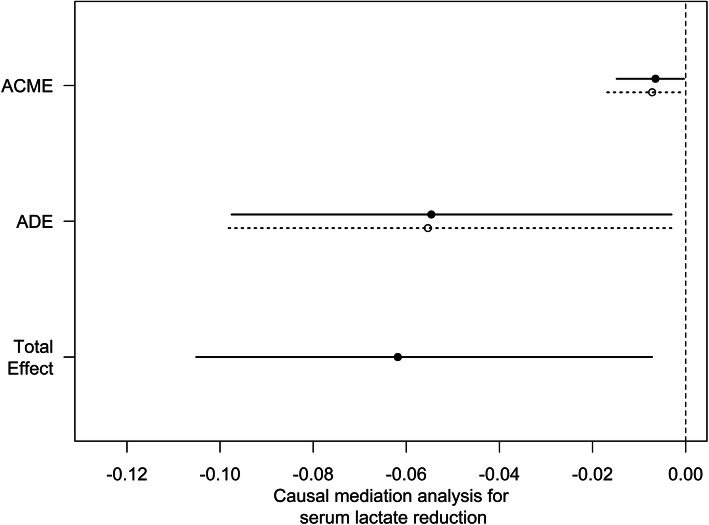
Causal mediation analysis for serum lactate reduction. The solid line represents the CVP group, and the dashed line represents the no CVP group

CMA showed that the reduction in serum lactate mediated 11% (95% CI 0.7%–66%; *p* = 0.04) of the beneficial effect of CVP measurement (*p* = 0.04 for ACME) in terms of 28-day mortality in septic patients (Fig. 4).

### Sensitivity analyses

The duration of CVP measurement did not alter the association (Additional file 1: Table S5). Regarding the initial CVP level, we first included patients with an initial CVP level below 8 mmHg in the CVP group and contrasted them against the no CVP group. The beneficial effect of CVP measurement (Additional file 1: Table S6) and the mediating effect of serum lactate reduction (*p* = 0.04 for the ACME) were similar to those in the main analysis. In addition, patients in the CVP group received more IVFs on day 1 and day 2 than those in the no CVP group (Additional file 1: Table S7). We then enrolled patients with an initial CVP level above 15 mmHg as the CVP group and reperformed the analyses. Although CVP measurement remained associated with a lower risk of 28-day mortality (Additional file 1: Table S8), the differences in the reduction in lactate (1.53 vs. 1.64 mmol/L; *p* = 0.543) and the mediation effect of serum lactate reduction (*p* = 0.08 for the ACME) were nonsignificant (Additional file 1: Table S9).

## Discussion

Our study demonstrated that CVP measurement was associated with significantly lower risk-adjusted 28-day mortality than no CVP measurement for the first time, as well as lower in-hospital and 1-year mortality, while no association with AKI within 7-days was detected. The mediating effect of serum lactate reduction on CVP measurement in terms of 28-day mortality was noticeable.

Although previous studies produced conflicting evidence concerning the impact of CVP on septic patients [19, 20], CVP has been widely used for more than 60 years to guide fluid therapy [21]. Understanding how CVP measurement influences clinicians’ decisions has improved over recent years. We abandoned targeting a specific CVP value due to the heterogeneity of patients; rather, we combined the trends of CVP and cardiac output to titrate fluid administration rather than rely on CVP alone. We realized that maintaining CVP as low as possible after initial haemodynamic stabilization was beneficial. We also extract valid information from CVP waves to aid clinicians [22]. However, the implications of CVP measurement on septic patient outcomes have never been questioned.

In our study, the SOFA score, SAPS II score, lactate level and incidence of AKI were significantly higher in the CVP group than in the no CVP group. Patients in the CVP group received mechanical ventilation and vasopressors more frequently than those in the no CVP group, and the MAP was lower in the CVP group than in the no CVP group. Despite these results, we found notably lower mortality in patients in CVP group than in those in the no CVP group after adjustment for confounding factors, and the relationship was robust regardless of the duration of CVP measurement and the initial CVP level. Consistent with limited results, in a nationwide, 1-day, prospective, point prevalence study, Machado et al. [1] declared that limited resources to treat sepsis (including the reduced availability of CVP measurements) were associated with increased mortality. In conclusion, our results highlight an essential role of CVP measurement in septic patients, and CVP measurement should not be abandoned at any time.

It was difficult to explore whether the therapeutic interventions were prompted by CVP measurement, and which interventions might account for the beneficial effects of CVP measurement in our retrospective observational study. CMA was applied to cover this limitation, and we hypothesized that CVP measurement-related triggers including fluid therapy could normalize lactate in septic patients with elevated lactate levels and further improve outcomes. In the present study, the volume of IVFs on day 1 and the reduction in serum lactate between day 1 and day 3 were higher in the CVP group than in the no CVP group. We used CVP measurement as the treatment and the reduction in serum lactate between day 1 and day 3 as a mediator variable and found that the effect of CVP measurement on 28-day mortality was proportionally mediated by the reduction in serum lactate.

CVP measurement-related triggers may be influenced by the initial CVP level. Eskesen et al. found a clear CVP level gradient in fluid responsiveness, at 75% at 0–5 mmHg, 55% at 6–10 mmHg, and 15% from 10 to 14 mmHg, with no patient responding to a level above 13 mmHg based on 1148 individual data sets [23]. We conducted sensitivity analyses to further understand the impact of the initial CVP level on the association and mediating effect described above. For patients in the CVP group with an initial CVP level below 8 mmHg, the volume of IVFs within 48 h after ICU admission and serum lactate reduction were higher than those in the no CVP group, and the mediating effect of serum lactate reduction was significant. The differences in serum lactate reduction and mediating effects were nonsignificant for patients in the CVP group with an initial CVP level above 15 mmHg. In line with these findings, a consensus statement recommended immediate fluid resuscitation in shock states associated with very low levels of preload parameters which including CVP [24]; an “extremely” high CVP level should not be used to predict or guide fluid resuscitation but may be used as a safety endpoint to avoid extrathoracic organ injury [22, 25, 26]. However, additional studies are needed to determine which interventions or protective factors mediate the beneficial effect of CVP measurement in patients, especially patients with an “extremely” high CVP level.

Our results simply reflect the true effect of CVP measurement in real-world clinical practice and confirm the benefit of CVP measurement in septic patients regardless of the initial CVP level. Clinicians should not abandon the measurement of CVP but consider how to utilize CVP in an appropriate way.

Several limitations in the present study should be considered. First, the definition of sepsis was based on infection and organ failure, but the infection diagnoses were undefined [27]. Hence, we included septic patients with positive blood cultures results in the subgroup analysis. Second, since the MIMIC-III data ranged from 2001 to 2015, the versions of the bundles might have changed during the period, and the results may not reflect current practices; hence, our results were adjusted for the admission period; in MIMIC-III database, we could not obtain the exact year of patient admission, and we divided patients into two groups in terms of admission time period (before 2008 and 2008–2012) for enrolment in the model. Third, there were multiple unmeasured confounders in our study that could affect our findings, such as different decisions by clinicians after CVP measurement and interventions before CVP measurement, and the use of other haemodynamic monitoring techniques in each group, including transthoracic echocardiography; pulmonary arterial catheters’ placement was unknown. Fourth, our study was a retrospective cohort study based on electronic healthcare records, the data generated during routine clinical visits. There are several technical challenges in measuring CVP, such as zero levels and reading errors, and the reliability of CVP levels in the present study is questionable. Fifth, when conducting the sensitivity analyses, except for the technical challenges, there are lots of factors that influence the initial CVP levels, including the initial PEEP, the expiratory phase when measured and the intraperitoneal pressure [8], and it is difficult to adjust these factors in a retrospective observational study. Finally, the causal relationship between CVP measurement and 28-day mortality was not explored thoroughly, and the reduction in lactate could have nothing to do with CVP measurement [28], as CVP measurement-related triggers are complex in clinical practice. A randomized study comparing the effect of CVP measurement and no CVP measurement is needed in the future.

## Conclusion

In conclusion, CVP measurement was associated with decreased risk-adjusted 28-day mortality in septic patients. The reduction in serum lactate may have mediated this effect, especially in patients with low CVP levels. In patients with high CVP levels, the mediating effect may be related to the reduced risk of extrathoracic organ injury.

## Supplementary information

**Additional file 1: Table S1.** Percentage of missing data in the variables of interest**. Table S2.** Association between CVP measurements and 28-day mortality with different models**. Table S3.** Analysis for patients with positive blood cultures (*n* = 4330). **Table S4.** Analysis for patients with septic shock (*n* = 2568). **Table S5.** Sensitivity analyses for patients with different duration of CVP measurements. **Table S6.** Sensitivity analysis for patients with an initial CVP level below 8 mmHg in CVP group**. Table S7.** Clinical outcomes after sensitivity analysis (CVP < 8 mmHg)**. Table S8.** Sensitivity analysis for patients with an initial CVP level above 15 mmHg in CVP group**. Table S9.** Clinical outcomes after sensitivity analysis (CVP > 15 mmHg)**. Figure S1.** Distribution of time to initial CVP measurements**. Figure S2.** Standardized mean difference (SMD) of variables before and after propensity score matching and weighting.

## Data Availability

The datasets presented in the current study are available in the MIMIC III database (https://physionet.org/works/MIMICIIIClinicalDatabase/files/).
