# Supplementary material for: Central venous pressure measurement is associated with improved outcomes in septic patients: an analysis of the MIMIC-III database
Source: Crit Care. 2020 Jul 14;24:433. doi: 10.1186/s13054-020-03109-9 (PMC7358999; doi:10.1186/s13054-020-03109-9)

**Table S1: Percentage of missing data in the variables of interest**

|  | MIMIC-III (n=10275) |
| --- | --- |
| Age | 0% |
| Male (%) | 0% |
| Weight (kg) | 8.7% |
| Service unit | 0% |
| Admission period | 0% |
| SOFA score | 0% |
| SAPS II score | 0% |
| Elixhauser comorbidity score | 0% |
| MV use (1^st^ 24 h) | 0% |
| Vasopressor use (1^st^ 24 h) | 0% |
| RRT use (1^st^ 24 h) | 0% |
| CHF | 0% |
| AFIB | 0% |
| Chronic renal disease | 0% |
| Liver disease | 0% |
| COPD | 0% |
| Stroke | 0% |
| Malignancy | 0% |
| MAP (mmHg) | 0.3% |
| Heart rate (bpm) | 0.3% |
| Temperature (℃) | 1.9% |
| Respiratory rate (bpm) | 0.38% |
| WBC (*10^9^/L) | 0.5% |
| Hemoglobin (*10^12^/L) | 0.38% |
| Platelet (*10^9^/L) | 0.44% |
| Bicarbonate (mmol/L) | 0.37% |
| Bun (mg/dl) | 0.2% |
| Creatinine (mg/dl) | 0.18% |
| Lactate level (mmol/L) | 29.4% |
| pH | 34.3% |
| pO_2_ (mmHg) | 34.3% |
| pCO2 (mmHg) | 34.3% |
| AKI, n (%) | 0% |
| 28-Day mortality | 0% |
| In-hospital mortality | 0% |
| 1-Year mortality | 0% |
| AKI within 7-day, n (%) | 0% |
| Volume of IVF on day 1(ml) | 4.9% |
| Volume of IVF on day 2(ml) | 15% |
| Volume of IVF on day 3(ml) | 37.6% |
| Vasopressor-free day in 28 days | 0% |
| Ventilation-free day in 28 days | 0% |
| Delta-lactate | 75.2% |

SOFA: sequential organ failure assessment; SAPS II: simplified acute physiology score II; MV: Mechanical ventilation; RRT: renal replacement therapy; CHF: congestive heart failure; AFIB: atrial fibrillation; COPD: chronic obstructive pulmonary disease; MAP: mean arterial pressure; WBC: white blood cell; PO_2_: partial pressure of oxygen; PCO_2_: partial pressure of carbon dioxide; AKI: acute kidney injury; IVF: intravenous fluid.

Table S2: Association between CVP measurements and 28-day mortality with different models

|  | Odds ratio | Lower 95% CI | | Upper 95% CI | | p value |
| --- | --- | --- | --- | --- | --- | --- |
| Multivariate model^*^ | 0.60 | | 0.51 | | 0.70 | <0.001 |
| Multivariate model after Multiple Imputation | 0.59 | | 0.51 | | 0.67 | <0.001 |
| PSM | 0.75 | | 0.64 | | 0.86 | <0.001 |
| IPTW | 0.73 | | 0.65 | | 0.84 | <0.001 |

*Multivariate model including age, sex, weight, service ICU, admission period, SOFA score, SAPS II score , use of mechanical ventilation, use of RRT, use of vasopressors, comorbidities, AKI, vital signs and initial lactate level.

PSM: Propensity score matching; IPTW: inverse probability of treatment weighing.

Table S3: Analysis for patients with positive blood cultures (n= 4330)

|  | Odds ratio | Lower 95% CI | Upper 95% CI | p value |
| --- | --- | --- | --- | --- |
| Multivariate model^*^ | 0.59 | 0.48 | 0.74 | <0.001 |
| Multivariate model after Multiple Imputation | 0.57 | 0.47 | 0.69 | <0.001 |
| PSM | 0.69 | 0.56 | 0.86 | <0.001 |
| IPTW | 0.69 | 0.57 | 0.82 | <0.001 |

*Multivariate model including age, sex, weight, service ICU, admission period, SOFA score, SAPS II score , use of mechanical ventilation, use of RRT, use of vasopressors, comorbidities, AKI, vital signs and initial lactate level.

PSM: Propensity score matching; IPTW: inverse probability of treatment weighing.

Table S4: Analysis for patients with septic shock (n=2568)

|  | Odds ratio | Lower 95% CI | Upper 95% CI | p value |
| --- | --- | --- | --- | --- |
| Multivariate model^*^ | 0.57 | 0.44 | 0.75 | <0.001 |
| Multivariate model after Multiple Imputation | 0.57 | 0.44 | 0.73 | <0.001 |
| PSM | 0.67 | 0.49 | 0.92 | 0.014 |
| IPTW | 0.75 | 0.60 | 0.92 | 0.02 |

*Multivariate model including age, sex, weight, service ICU, admission period, SOFA score, SAPS II score , use of mechanical ventilation, use of RRT, use of vasopressors, comorbidities, AKI, vital signs and initial lactate level.

PSM: Propensity score matching; IPTW: inverse probability of treatment weighing.

Table S5: Sensitivity analyses for patients with different duration of CVP measurements

|  | Odds ratio* | Lower 95% CI | Upper 95% CI | p value |
| --- | --- | --- | --- | --- |
| ≤ 1 day | 0.51 | 0.38 | 0.69 | <0.001 |
| ≤ 2 days | 0.57 | 0.46 | 0.70 | <0.001 |
| ≤ 3 days | 0.52 | 0.43 | 0.63 | <0.001 |
| ≤ 4 days | 0.53 | 0.44 | 0.63 | <0.001 |
| ≤ 5 days | 0.55 | 0.46 | 0.66 | <0.001 |
| ≤ 6 days | 0.58 | 0.49 | 0.68 | <0.001 |
| ≤ 7 days | 0.58 | 0.49 | 0.69 | <0.001 |
| > 7 days | 0.64 | 0.51 | 0.80 | <0.001 |

*Multivariate model including age, sex, weight, service ICU, admission period, SOFA score, SAPS II score , use of mechanical ventilation, use of RRT, use of vasopressors, comorbidities, AKI, vital signs and initial lactate level.

Table S6: Sensitivity analysis for patients with an initial CVP level below 8 mmHg in CVP group

|  | Odds ratio | Lower 95% CI | Upper 95% CI | p value |
| --- | --- | --- | --- | --- |
| Multivariate model^*^ | 0.56 | 0.45 | 0.69 | <0.001 |
| Multivariate model after Multiple Imputation | 0.54 | 0.45 | 0.66 | <0.001 |
| PSM | 0.65 | 0.53 | 0.80 | <0.001 |
| IPTW | 0.67 | 0.56 | 0.80 | <0.001 |

*Multivariate model including age, sex, weight, service ICU, admission period, SOFA score, SAPS II score , use of mechanical ventilation, use of RRT, use of vasopressors, comorbidities, AKI, vital signs and initial lactate level.

PSM: Propensity score matching; IPTW: inverse probability of treatment weighing.

Table S7: Clinical outcomes after sensitivity analysis (CVP < 8 mm Hg)

|  | CVP | NO CVP | Effect size | P value |
| --- | --- | --- | --- | --- |
| **Primary outcome** | | | | |
| 28-Day mortality | 198/1119 (17.7) | 277/1119 (24.8) | 0.173 | <0.001 |
| **Secondary outcomes** | | | | |
| In-hospital mortality | 183/1119 (16.4) | 235/1119 (21) | 0.119 | 0.005 |
| 1-Year mortality | 419/1119 (37.4) | 488/1119 (43.6) | 0.126 | 0.003 |
| AKI within 7-day, n (%) | 867/1119 (77.5) | 850/1119 (76) | 0.036 | 0.395 |
| Volume of IVF on day 1(ml) | 2900 (1578.5-4883.8) | 1959.2 (900-3063) | 0.485 | <0.001 |
| Volume of IVF on day 2(ml) | 1251 (347-2436.3) | 1050 (260-2004.8) | 0.246 | <0.001 |
| Volume of IVF on day 3(ml) | 691.8 (247.5-1673.5) | 734 (240-1729) | 0.041 | 0.737 |
| Vasopressor-free day in 28 days | 26.8 (25.4-27.5) | 26.3 (23.6-27.2) | 0.408 | <0.001 |
| Ventilation-free day in 28 days | 26.2 (22.9-27.2) | 24.1 (17.8-26.9) | 0.295 | <0.001 |
| Delta-lactate | 1.75 (2.23) | 1.28 (2.44) | 0.199 | 0.035 |

IVF: intravenous fluid; AKI: acute kidney injury.

Table S8: Sensitivity analysis for patients with an initial CVP level above 15 mmHg in CVP group

|  | Odds ratio | Lower 95% CI | Upper 95% CI | p value |
| --- | --- | --- | --- | --- |
| Multivariate model^*^ | 0.71 | 0.56 | 0.89 | 0.003 |
| Multivariate model after Multiple Imputation | 0.63 | 0.51 | 0.77 | <0.001 |
| PSM | 0.71 | 0.56 | 0.90 | 0.004 |
| IPTW | 0.71 | 0.58 | 0.87 | 0.001 |

*Multivariate model including age, sex, weight, service ICU, admission period, SOFA score, SAPS II score , use of mechanical ventilation, use of RRT, use of vasopressors, comorbidities, AKI, vital signs and initial lactate level.

PSM: Propensity score matching; IPTW: inverse probability of treatment weighing.

Table S9: Secondary outcomes after sensitivity analysis (CVP > 15 mmHg)

|  | CVP | NO CVP | Effect size | P value |
| --- | --- | --- | --- | --- |
| **Primary outcome** | | | | |
| 28-Day mortality | 165/793 (20.8) | 214/793 (27) | 0.145 | 0.004 |
| **Secondary outcomes** | | | | |
| In-hospital mortality | 173/793 (21.8) | 197/793 (24.8) | 0.072 | 0.154 |
| 1-Year mortality | 316/793 (39.8) | 346/793 (43.6) | 0.077 | 0.127 |
| AKI within 7-day, n (%) | 703/793 (88.7) | 679/793 (85.6) | 0.090 | 0.072 |
| Volume of IVF on day 1(ml) | 2132.5 (792-4135.6) | 1750 (778-3000) | 0.238 | <0.001 |
| Volume of IVF on day 2(ml) | 840 (288-2100) | 810 (250-1904) | 0.161 | 0.078 |
| Volume of IVF on day 3(ml) | 596 (240-1500) | 625 (240-1635) | 0.090 | 0.981 |
| Vasopressor-free day in 28 days | 26.6 (25-27.4) | 25.5 (21.1-27.0) | 0.483 | <0.001 |
| Ventilation-free day in 28 days | 26.0 (22.4-27.2) | 22.4 (14.9-26.2) | 0.601 | <0.001 |
| Delta-lactate | 1.53 (1.67) | 1.64 (1.94) | 0.057 | 0.543 |

IVF: intravenous fluid; AKI: acute kidney injury.

Figure S1: Distribution of time to initial CVP measurements


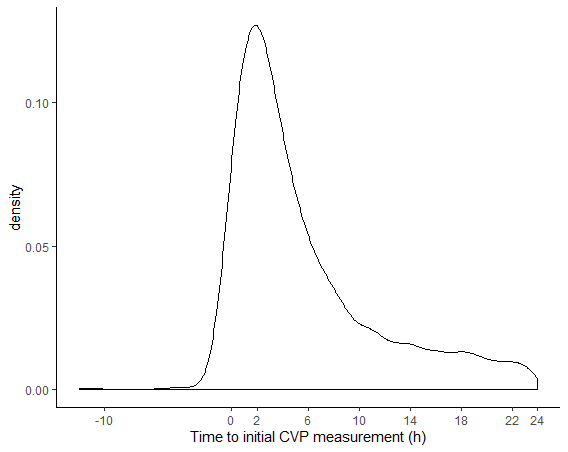


Figure S2: Standardized mean difference (SMD) of variables before and after propensity score matching and weighting


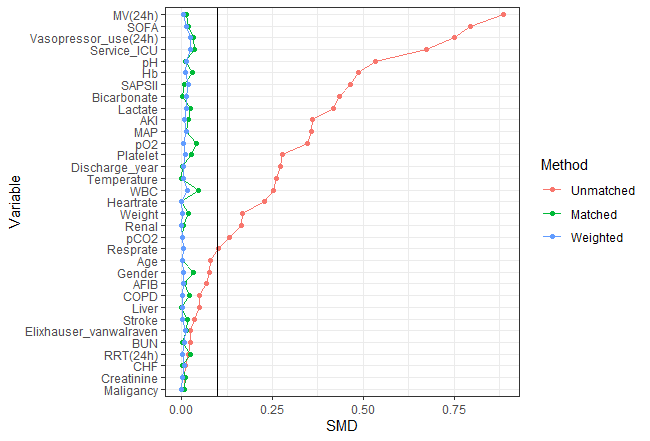

Supplement: Supplementary file 1 — Additional file 1: Table S1. Percentage of missing data in the variables of interest. Table S2. Association between CVP measurements and 28-day mortality with different models. Table S3. Analysis for patients with positive blood cultures (n = 4330). Table S4. Analysis for patients with septic shock (n = 2568). Table S5. Sensitivity analyses for patients with different duration of CVP measurements. Table S6. Sensitivity analysis for patients with an initial CVP level below 8 mmHg in CVP group. Table S7. Clinical outcomes after sensitivity analysis (CVP < 8 mmHg). Table S8. Sensitivity analysis for patients with an initial CVP level above 15 mmHg in CVP group. Table S9. Clinical outcomes after sensitivity analysis (CVP > 15 mmHg). Figure S1. Distribution of time to initial CVP measurements. Figure S2. Standardized mean difference (SMD) of variables before and after propensity score matching and weighting. [file 13054_2020_3109_MOESM1_ESM.docx]
